# Treatment of Palatally Displaced Canines in Children: A Randomized Controlled Pilot Trial on Exposure Time and Patient Perception of Two Closed Surgical Methods

**DOI:** 10.1002/cre2.70233

**Published:** 2025-10-13

**Authors:** Katja Hashemi Elses, Krister Bjerklin, Ann‐Marie Roos Jansåker, Mikael Sonesson

**Affiliations:** ^1^ Department of Orthodontics, Faculty of Odontology Malmö University Malmö Sweden; ^2^ Department of Periodontology, Faculty of Odontology Malmö University Malmö Sweden

**Keywords:** closed exposure technique, impacted canines, orthodontics, surgical exposure

## Abstract

**Objectives:**

To evaluate treatment time and patient perception of two surgical methods to expose a palatally displaced canine (PDC) into the oral cavity.

**Material and Methods:**

A total of 30 consecutive patients between 11 and 18 years, with maxillary displaced canines were recruited. After gaining informed consent from the patients and custodians, the patients were randomized into two groups by an independent person. Both groups received a chain attached to the crown of the canine: in group A (control group) the chain was placed under the mucoperiosteal flap to an incision on the alveolar crest and in group B (test group), the chain penetrated the mucoperiosteal flap inferiorly to the crown of the canine. Outcome measures where time to expose the PDC into the oral cavity and the patient's experience of pain and discomfort during the treatment.

**Results:**

Twenty‐six patients full‐filled the trial, mean age was 12.9 years, (SD 1.6 years). The time to expose the canines for the control group was 11.9 months (SD 6.5) and for the test group 6.7 months (SD 3.2) The conventional method showed less pain on the day of surgery.

**Conclusion:**

The method used in the test group resulted in a 5‐month shorter time to expose the canine compared to the control group, and higher pain level on the day of surgery. For generalizability of the results, larger studies are needed.

## 1 Introduction

The maxillary canine is important for the development of a normal occlusion, and, in most children, the canine erupts uneventfully. However, in some children, canines are impacted buccally, palatally or within the dental arch. The frequency of maxillary impacted canines is 2%–3% and seems to be more common in females than males (Thilander and Myrberg 1973). Earlier investigations have shown that 40%–85% of the patients with displaced canines have a palatally position and 8% have a bilaterally displacement of the canines (Thilander and Myrberg 1973; Dachi and Howell 1961; Jacoby 1983; Hou et al. 2010).

The etiology of eruption disorders is not fully understood but a genetic predisposition or lack of eruption guidance is suggested (Jacoby 1983; Becker et al. 1981).

The risk of damage to the adjacent roots is imminent. Previously, researchers showed that up to 50% of the patients with impacted maxillary canines have root resorption of the adjacent incisors (Ericson and Bjerklin 2001; Ericson and Kurol 2000).

The diagnostics and treatment of ectopic or impacted maxillary canine is one of the most time‐consuming orthodontic treatments and commonly includes extended radiological examinations, surgery and orthodontic treatments (Stewart et al. 2001). The surgery and the traction of the maxillary canine relate to discomfort and pain for the patients (Björksved et al. 2021; Gharaibeh and Al‐Nimri 2008).

Usually, the affected canine is exposed by a closed or open surgical technique and sometimes modified to improve eruption and reduce treatment time, as when applying glass ionomer on the canine during open exposure (Naoumova et al. 2018). A systematic review concluded previously that there is limited scientific evidence on the effectiveness of different methods to treat impacted maxillary canines and a need of clinical research investigating treatment options to create evidence based clinical guidelines (Grisar et al. 2021).

Lately, the patient reported outcome measures (PROM) during the exposure of the canines as well as different treatment techniques to move the canine, have been investigated, but the study designs are heterogenous with different generic questionnaires, which makes it difficult to draw any conclusions (Becker et al. 2016; Sampaziotis et al. 2018; Björksved et al. 2018).

During treatment with closed exposure, the chain sometimes interferes with the healing process, which results in pain during traction and even sometimes increases the need of additional radiological examinations and re‐exposure. To reduce the unwanted side‐effects, a modified closed technique has been developed in the clinic where the chain penetrates the mucoperiosteal flap inferiorly to the palatal impacted canine instead of running in the submucosa to an incision on the alveolar crest. The placement intends to facilitate the control of the chain and the tooth.

To our knowledge, no study investigating differences in treatment time and PROM between a modified method, with an incision around the crown of the canine and a conventional method, with an incision on the alveolar crest, to treat the ectopic or impacted canines, seems available.

The overall aims of the present pilot trial were to compare treatment time for exposure of the canine into the oral cavity, and the patient related treatment outcome using; (A) a conventional closed exposure method or (B) a modified method. The null hypotheses were no differences, in time to expose the canine or in pain and discomfort between the two methods.

## 2 Materials and Methods

### 2.1 Study Design

The trial was carried out at the orthodontic clinics in Kristianstad and Helsingborg, in Sweden, and was designed as a randomized, single‐blinded controlled pilot trial with two parallel arms. The study was ethically approved by the Sweden Ethical Review Authority (Dnr.2013/93, 2023‐07096‐02) following the guidelines of the Declaration of Helsinki.

### 2.2 Patients and Eligibility Criteria

A total of 35 eligible patients were invited to participate in the study and 30 patients, 19 females and 11 males, accepted to be enrolled. The patients were consecutively recruited between January 2013 and May 2019. The inclusion criteria were healthy patients between the ages of 11 and 18 years with palatally displaced canines (PDC). The canines should be positioned in sectors 2–5 with an alfa angle between 10° and 75°, on a panoramic radiograph according to Ericson and Kurol (1988). No difference in treatment or special consideration was made to patients with bilaterally impacted maxillary canines.

Exclusion criteria were patients with clefts, syndromes, or previous orthodontic treatment.

### 2.3 Allocation and Randomization

All patients and their legal guardians were given verbal and written information by one orthodontist (KHE) and had to sign an informed consent form before the allocation process. Opaque envelopes with papers signing the group affiliation, group A (control group) or group B (test group), were prepared. The envelopes were sequentially numbered and sealed separately by an independent assistant not involved in the trial or the treatments. The envelopes were kept securely at the clinic and not available for anybody in the research team. Three independent dental nurses, not involved in the surgery, were in charge for the envelopes and familiar with randomization process. In the beginning of the surgical session, when the mucosal flap was raised, one of the nurses, who stayed in close proximity to the operating room and had access to the numbered envelopes, was called. Because of the sterile conditions in the room, the nurse picked an envelope and revealed the group assignment by opening the sealed envelope. The patients in group A were treated according to the conventional method for closed exposure technique and the patients in group B were treated according to the modified method with the chain penetrating the mucosa inferiorly to the canine crown.

### 2.4 Blinding

Because of the nature of the trial, neither the participants nor the operators were blinded. However, the person who analyzed the statistical data was blinded to the group assignments.

### 2.5 Clinical Examination and Registration

Before the treatment started, a regular orthodontic clinical examination and registration of the patient was performed. The registration included plaster casts and five intra‐ and six extra‐oral digital photographs (Nikon D7100, Macro Speedlight SB‐21, Japan). Three intra‐oral periapical radiographs of the anterior maxilla and panoramic radiographs (Planmeca, Pro X, Asentajankatu 6, FI‐00990, Helsinki, Finland). Lateral radiograph and Cone beam computed tomography (CBCT) investigations were performed on individual indications.

### 2.6 Surgery

**Figure 1:**
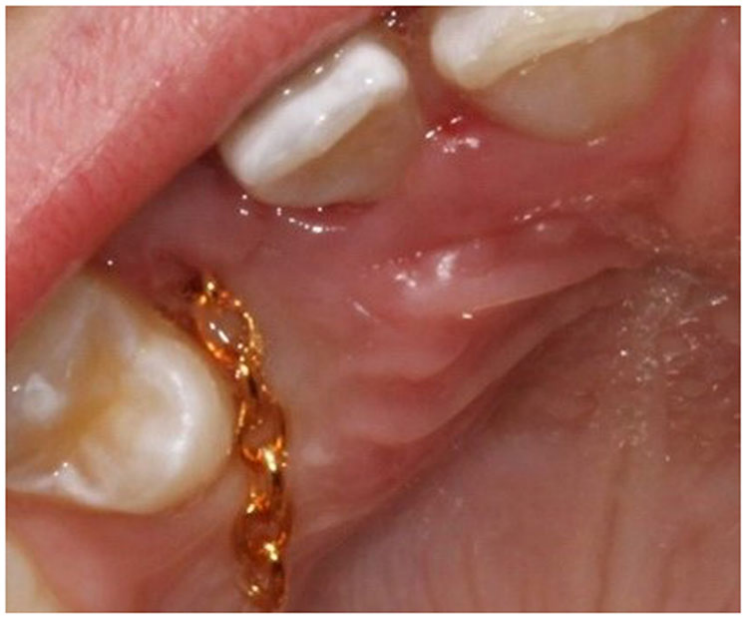
Group A (control group), the chain runs under the mucoperiosteal flap and enter the oral cavity through an incision on the alveolar crest.

**Figure 2:**
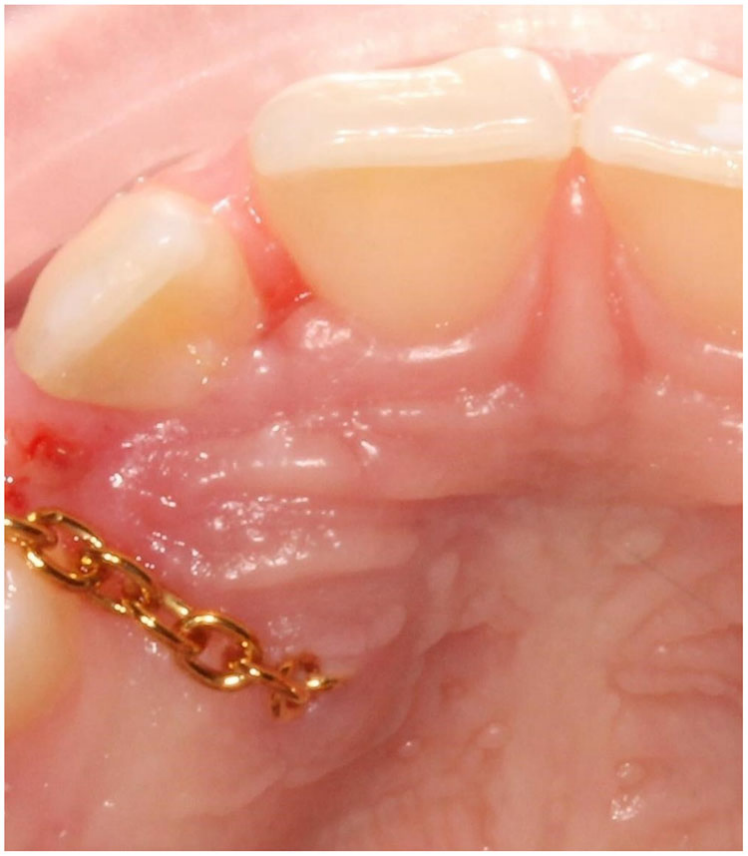
Group B (test group), the chain penetrates the mucosa inferiorly to the crown of the canine through an incision in the mucoperiosteal flap.

The surgery was performed at the department of periodontology in xx and yy by one senior consultant periodontist (AMRJ). After anesthesia, Xylocain Adrenalin (10 mg/mL; 3.6 mL–5.4 mL), a sulcular incision was made at the palatal site of the teeth in the region of the impacted canine and extended to raise a flap with access to the area of the crown of the canine. Bone and follicular tissues were removed, and the crown was exposed. The canine was luxated with a light force and a chain (Stainless Steel Traction Chain with Eyelet Button Chain Dental, BroTech, Sweden) was bonded to the crown (Ketac Fil Plus 3 M, Sweden). In group A (control group), the chain was positioned under the mucoperiosteal flap and entered the oral cavity through an incision on the alveolar crest (Figure 1). In group B (test group), an incision was made in the flap providing the chain to penetrate the mucosa inferiorly to the crown of the canine (Figure 2). The chain was then connected to the orthodontic appliance. After profuse rinsing with a saline solution, the area was sutured with non‐resorbable sutures (Supramide 4‐0, DS19, B. Braun, Swemed, Sweden). The patient was informed after surgery to rinse with chlorhexidine (0.2%) until the next appointment approximately 10–14 days later when the sutures were removed, and the chain was activated.

### 2.7 Orthodontic Treatment

The patients were treated by one senior consultant in orthodontics (KHE) serving at the two orthodontic clinics, using pre‐adjusted fixed appliances in the maxilla and the mandible (.022 slot size, MBT prescription, Victory Series, 3M Unitec, Monovia, CA, USA). The fixed appliances were installed 3 weeks before the surgical exposure. All patients received a fixed transpalatal bar (MR Bands, AO, USA) attached to the maxillary first permanent molars.

Two weeks after the surgery, the orthodontic traction started. In both groups, a traction elastic string (Slip Free Zing O String .025 mm, TP Ortho Inc.) was attached between the chain to the canine and the orthodontic appliance.

Activation of the orthodontic elastic string was performed every 6 weeks at the specialist clinic by one orthodontist (KHE). The direction of the traction followed a protocol, used for both group A and group B (Appendix 1).

When the canine was exposed to the oral cavity, the tooth was bonded with an orthodontic bracket and guided in the direction to its normal dental arch position by the zing‐string traction.

### 2.8 Registration Protocol After Surgical Exposure

If the canine was not clinically exposed in the oral cavity after 5 months or it was assumed that the attachment on the canine was detached, two standardized intraoral radiographs were obtained. Two additional standardized intraoral radiographs were obtained every fifth month until the canine was finally exposed to the oral cavity. Digital intraoral photographs were obtained when the crown of the canine was exposed in the oral cavity.

### 2.9 Questionnaires

The questionnaires were adopted from a previous clinical trial on measurements of patients' post‐operative pain and pain during orthodontic treatment (Feldmann et al. 2007). The questionnaire includes a VAS scale from 0 to 10 with the end phrases no pain at all and worst imaginable pain. The questionnaires were answered as follows: (1) on the same evening as the surgery, (2) 1 week after the surgery, and (3) every 3 months until the canine erupted. The VAS scale was grouped into three categories during the analysis of the data, 0–3, 4–6, and 7–10, corresponding to low, medium, and high pain (Zieliński et al. 2020; Jensen 2003).

### 2.10 Primary Outcome Measurements

The primary outcome measurements were treatment time measured from the surgery to exposure of the maxillary canine in the oral cavity as well as positioned in the dental arch.

### 2.11 Secondary Outcome Measurements

The secondary outcome measurements were pain and discomfort assessed on the day of surgery, 7 days after surgery, and during the traction of the canines until the canine was exposed to the oral cavity.

### 2.12 Sample Size Calculation

No sample size calculation was used. It has been suggested in the literature that 12 subjects in each arm might be sufficient to reach precision of the mean and the variance, and to perform a feasible study (Julious 2005). Thus, we decided to recruit 15 subjects in each arm to compensate for dropouts.

### 2.13 Statistical Analysis

Normality in distribution was tested by Shapiro–Wilks test. *T*‐test was used for independent normal distributed variables, and the Mann–Whitney *U*‐test was used for independent variables that were not normally distributed.

## 3 Results

**Figure 3:**
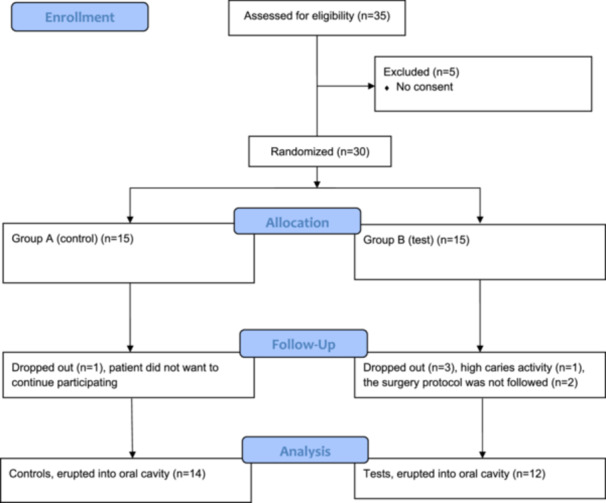
Flow chart for participants and dropouts in the trial.

A total of 26 patients completed the protocol for exposure into the oral cavity, 14 in group A (control group) and 12 in group B (test group). The mean age of the patients before treatment was in group A, 12.4 years (SD 0.93) and in group B, 13.7 years (SD 1.97) (Table 1). In group A, one patient did not want to continue the orthodontic treatment. The canine was then surgically removed. In group B, one patient did not comply with the oral hygiene and was assessed as highly caries active and debonded. Finally, two patients were excluded because of failure to follow the surgery protocol. A flow‐chart with the reasons for dropping out is presented in Figure 3.

**Table 1:** Sample characteristics.

	Patients	Group A	Group B	
	*n* = 26	*n* = 14	*n* = 12	
Age (years)	12.96 (1.61)	12.36 (0.93)	13.67 (1.97)	0.068^a^
Mean (SD)	12.00 (11.00–18.00)	12.00 (11.00–14.00)	13.00 (12.00–18.00)	
Median (min–max)				
Gender, *n* (%)				
Male	10 (38.5)	5 (35.7)	5 (41.7)	0.756^b^
Female	16 (61.5)	9 (64.3)	7 (58.3)	
Sector, *n* (%)				
Sector 2	6 (23.1)	5 (35.7)	1 (8.3)	0.449^b^
Sector 3	11 (42.3)	7 (50.0)	4 (33.3)	
Sector 4	8 (30.8)	2 (14.3)	6 (50.0)	
Sector 5	1 (3.8)	0	1 (8.3)	
Angele				
< 45°	20 (76.9)	13 (92.9)	7 (58.3)	0.195^b^
> 45°	6 (23.1)	1 (7.1)	5 (41.7)	

*Note: n* (%), number of patients (percentage).

^a^ Mann–Whitney *U* test, 2‐sided.

^b^ Pearson *χ* ^2^ test, 2‐sided.

In group A, five PDC were in sector two, seven PDC in sector three, and two PDC in sector four. In group B, one PDC was in sector two, four in sector three, six in sector four, and one in sector five (Table 1). The mean alfa angle was 32.3° in group A and 45.3° in group B, the difference was statistically significant.

In four patients, three in group A and one in group B, extraction of the first maxillary premolar was necessary because of maxillary crowding. The extractions were performed during the surgical exposures of the canine. All 26 patients who remained in the trial had a successful eruption of the PDC into the oral cavity. A total of 14 patients had impacted maxillary canines bilaterally, nine in group A and five in group B. The bilateral impacted canines were displaced palatally, buccally or central in the alveolar crest.

### 3.1 Treatment Time

The treatment time for the canines to be exposed to the oral cavity was in group A 11.9 months and in group B 6.7 months. The difference was statistically significant (*p* = 0.02) (Table 2).

**Table 2:** Treatment time (months), number of patients (*n*).

	Group A	Group B	
Eruption time			
*n* mean (SD) Arch position time *n* mean (SD)	14 11.9 (6.5) 14 8.7 (4.4)	12 6.7 (3.2) 9 11.8 (8.0)	0.020^a^ > 0.05

Abbreviation: SD, standard deviation.

^a^ *p* value: Independent samples *t*‐test, two‐tailed.

The time between exposure of the canine into the cavity and to positioning the canines in the dental arch was in group A 8.7 months and in group B 11.8 months (Table 2). No statistically significant difference between the groups was found.

### 3.2 Pain and Discomfort

No impact on daily activities was registered nor was functional jaw impairment observed during the evening after surgery in any of the two groups. None of the patients needed to stay at home from activities or school because of the surgery on the same day of surgery or 7 days afterwards. The patients in group B (test group) experienced higher levels of pain and discomfort when chewing soft food (question 13, Appendix 2) compared to group A (control group) in the evening of the day of surgery and chewing hard food 7 days after surgery (question 19, Appendix 2). The differences were statistically significant (*p* ≤ 0.05).

In total, 22 patients used one Paracetamol tablet (500 mg) on the evening of the surgery, and four patients continued to use Paracetamol for one additional week if they experience pain or discomfort. Three patients used Paracetamol for 3 months if necessary. The intake of analgesics was equally distributed between group A and B. Eleven patients were given 10 mL sedation (Midazolam APL peroral 1 mg/mL) before surgery, three in group A and eight in group B. No differences between the groups in pain and discomfort were seen 7 days after the surgery except for the site of injection, where group B reported higher pain 1‐week post‐surgery compared to group A. The difference was, however, not statistically significant.

### 3.3 Complications During Orthodontic Traction

Two re‐exposures were needed in group A and in group B, one because of detachment of the chain and one because of interference between the chain and the healing process. Thus, no significant differences in the total number of complications between the groups were observed.

## 4 Discussion

### 4.1 Main Findings

In this randomized controlled pilot trial, a statistically significant shorter time to expose the maxillary displaced canines in the oral cavity was seen in the test group (group B) having the traction chain through the mucosa close to the canine, compared to the control group (group A) (*p* = 0.02). The patients in the test group experienced significantly more pain and discomfort on the same evening as the surgery compared to the controls. Thus, our null hypothesis is partly rejected.

Previously it was shown that the total treatment time varies due to the canine's position, angulation, tissue responses to traction, and the patient´s compliance with the treatment (Björksved et al. 2021; Grisar et al. 2021; Sampaziotis et al. 2018)

In the present study, the time to expose the canines in the oral cavity was 5.2 months shorter in the test group (group B) compared to the control group. There might be an uncertainty of the results because of existing heterogeneity between the groups in sector position and angle to midline of the PDC as well as the number of bilaterally PDCs. Two patients in the control group had a PDC in sector four and six patients in the test group had a PDC in sector four and one in sector five. Björksved and co‐authors (2019) showed however that the PDC sector position and angle to midline were assessed as more severe in panoramic radiographs compared to CBCT. This resulted in fair agreements between the two radiograph methods. Thus, the clinical differences between the positions of the PDCs in the two groups might not be as large as the differences assessed on the panoramic radiographs. However, the results should be interpreted with some caution because the uncertainty of the size of the impact of the present differences between the groups.

The significant difference between the groups in treatment time can rather be explained by better control of the traction components as the chain is easier to monitor and also creates less interaction with the healing processes, compared to the controls having the chain embedded into the tissues. It seems reasonable that this initial part of the treatment becomes shorter, which might decrease the treatment costs. This needs to be investigated in future trials.

No statistically significant differences between the groups were detected concerning the time to move the canines into the dental arch. One explanation might be that the canines in the test group are exposed in the cavity more distant to the final position in the dental arch, thus additional time is needed to position the teeth. In addition, due to lack of compliance of one patient in the test group (group B) to visit the clinic, the canine was moved more posteriorly than planned, which took additional time to correct. However, once the canine is visible in the oral cavity, the movement should be easier to follow, which facilitates the treatment.

### 4.2 Radiological Methods

In the present study, we used panoramic radiographs to describe sectors and angulation of the PDC according to the previous reports of Björksved and co‐workers and the study of Ericson and Kurol (1988). However, Björksved and co‐workers (2019) showed that the assessments on panoramic radiographs resulted in an overestimation of the displacement of the canine compared to intraoral periapical radiographs and CBCT. The assessed canine on panoramic radiographs also appears to be closer to the maxillary midline than the canine really is. When comparing the position of the canines on intraoral periapical radiographs and CBCT, Haney et al. (2010) showed that CBCT offers more reliable diagnostics, leading to potentially better treatment outcomes for the patients.

However, the method to use the panoramic radiographs for assessment of the position and angulation of PDC is an accepted method both in the clinic and in research, which makes the results comparable with other studies. But the method is not validated which might increase the risk of performance bias in our study.

The use of CBCT was indicated on individual reasons in 16 patients to assess signs of external root resorption of the lateral incisors and the position of the canines. The indications seem to be similar to other reports concerning CBCT during the treatment of impacted canines (Björksved et al. 2019).

### 4.3 Pain and Discomfort

In general, patient perceptions of pain and discomfort are affected by several factors, such as pre‐surgery analgesics, sedation, gender, age, previous experiences of dental care according to Zabielskaite et al. (2022), there is no significant relation between the discomfort and the location of the impacted tooth. In present study, the patients in the test group reported higher discomfort scores in the evening of the day of surgery compared to the controls. The difference might be explained by the location of the incision of the mucoperiosteal flap, inferiorly to the crown, which certainly is in contact with the bolus when the patient chews. The patient's perception of pain might also be influenced by the severity of the surgery and the higher number of patients with a PDC in a more severe position in the test group might have influenced the pain perception results. In five patients one premolar was also extracted during the surgery, which might have influenced the feeling of pain after surgery. However, the patients who experienced extractions were almost equally distributed between the groups. In addition, extraction of premolars has been shown to be well‐tolerated by patients with moderate pain 2 h after extraction (Berlin et al. 2019). The use of generic questionnaires for the assessment of pain and discomfort is more often examined in orthodontic investigations today but there is a need of additional tools to perform patient‐centered assessments (Perazzo et al. 2020; Krekmanova et al. 2009). Qualitative methods to investigate patient related outcomes seems to be rare in orthodontic investigations. However, the use of a qualitative method with in‐depth interviews or focus groups might have resulted in a more valid description of the patient perceptions in the two groups of patients in this study.

In total, 24 of 30 patients used analgesics the same evening as the surgery and six patients continued using analgesics for 1 week. The results indicate that most of the patients seem to need analgesics to reduce pain during the day of surgery. Recently, it has been suggested that dentists should prescribe analgesics to reduce post‐surgery pain more effectively, which seems reasonable according to our results (Zieliński et al. 2020). In the present study, however, the need for analgesics during orthodontic traction seems to be rather low, as just three patients continued using Paracetamol for more than 1 week.

### 4.4 Surgery Procedure

The assessed time for the surgery procedure did not differ between the two methods. In both groups, a mucoperiosteal flap was raised and the bone and dental follicle were removed before the clinical crown of the canine was exposed. The additional incision on the alveolar crest in the test group (group B), did not have an impact on the surgery procedure time.

The rate of complications such as detachments, infections with prescription of antibiotics, and re‐exposure has been reported to be approximately 9% in treatments of displaced canines (Parkin et al. 2012). In the present study, no emergency visit because of infection, bleeding or swelling was registered after surgery and no prescription for antibiotics was registered. In two patients, a re‐exposure of the canine was needed, and these exposures were equally distributed between the groups and of limited severity. The low number of complications in the present study might be explained by the fact that all surgical exposures were performed by a single experienced operator.

### 4.5 Strengths and Limitations

A strength of the present study is the design with a randomized clinical trial with two parallel arms.

The randomization was performed by an independent person who opened the envelope expelling if the patient would belong to group A or group B. This procedure was performed at the time when the flap was raised, and the surgeon could now continue the operation for a group A or group B patient, which should reduce the risk of selection bias.

One limitation of our study was the small sample size, which had an impact on the mean age of the groups; one patient in the test group was 18 years old. The small sample size might also influence the results of the evaluation of the patient's pain and discomfort. However, the grouping of the VAS scale into three categories, low, medium and high levels of pain and discomfort, increases the validity of the questionnaires and the high response rate increases the representativeness of the results. Another limitation was that the number of patients was further reduced due to the covid pandemia which meant that we were not able to assess the time for the whole treatment until the fixed appliances were removed. However, in 25 patients we also assessed the time to move the canines into the dental arch. The time did not differ between the groups which indicates that the primarily advantage of the new method is a faster exposure of the canines, but then the movement to the dental arch appears to proceed similarly in the two methods. This trial was designed as a pilot study and the number of patients included is relatively low. However, there seems to be no consensus about the sample size in pilot trials and between 8 and 114 subject per arm has been reported (Billingham et al. 2013).

Another limitation is the heterogenicity of patient groups. In 14 patients, nine in the control group and five in the test group, both the maxillary canines were impacted. The canines with the most severe positions were included in the trial as these teeth were urgent to treat as fast as possible to avoid damage to the patients. The treatment outcomes of the second canines were not evaluated. The differences between the groups in number of patients with bilaterally canines might have an impact on the total treatment time but investigating the treatment time and patient's perception with bilaterally PDC was out of the scope of this study and the number of patients in the subgroups were too low, especially for assessment of patient perception. However, the impact of the higher number of patients with severe PDC positions in the test group and the higher number of bilaterally PDCs in the control group is a variable to consider when planning clinical treatment trials of patients with PDC.

Finally, by using a per‐protocol approach, analyzing only participants who were compliant with the study protocol, increases the risk of reporting bias and an over‐optimistic estimation of the efficacy, compared to an intention‐to‐treat (ITT) approach. However, the number of patients included in the present study was too small for an ITT analysis (Ahn and Kang 2023; Wright and Simb 2002).

## 5 Conclusion

The treatment time of the test method to get the PDC exposed in the oral cavity by moving the incision from the alveolar crest to the area of the crown, was 5 months shorter compared to the control method. The patients treated by the test method showed more pain when chewing during the day of surgery. Patients in both groups used analgesics on the day of surgery, and just a few patients used analgesics for a longer period. New multi‐center randomized controlled trials on effectiveness, patient related outcome measures, and costs of the total treatment of palatally displaced canines, from exposure to debonding, are needed to create evidence‐based clinical recommendations in the orthodontic care.

## Author Contributions

All authors contributed to the study conception and design. Material preparation, data collection, and analysis were performed by xx and yy. The first draft of the manuscript was written by xx and uu, all authors commented on previous versions of the manuscript. All authors have read and approved the final manuscript.

## Ethics Statement

No unexpected harm was observed in any subject of the included two groups of patients.

## Conflicts of Interest

The authors declare no conflicts of interest.

## Clinical Trial Registration

The study commenced before a discussion on registration in Clinical Trials. gov, thus the study could not be registered.

## Supporting information

Appendix: Surgical Exposure Questionnaire.

supp.

supp.

supp.

## Data Availability

The data set generated and analyzed in present study is not publicly presented due to ethical reason but is available from the corresponding author upon reasonable request.
