# Supplementary material for: Treatment of Palatally Displaced Canines in Children: A Randomized Controlled Pilot Trial on Exposure Time and Patient Perception of Two Closed Surgical Methods
Source: Clin Exp Dent Res. 2025 Oct 13;11(5):e70233. doi: 10.1002/cre2.70233 (PMC12516783; doi:10.1002/cre2.70233)
Supplement: Supplementary file 1 — Appendix: Surgical Exposure Questionnaire. [file CRE2-11-e70233-s001.docx]

**Protokoll Retinerade Hörntänder palatinalt ök**

**# Registreringar före behandlingsstart**

Registreringsdatum…………………

Namn…………………………………………………. Födelsenr………………………. Löpnummer……….

Kön…………. 1=pojke, 2=flicka

**---------------------------------------------------------------------------------------------------------------**

Tand………… 1 = 13, 2 = 23 endast enkelsidiga

Agenesi lateralen………… 1= ja, 2= nej

Behandlingsplan ortodonti…………… 1= starta med TPB med utlöpare eller sekt båge, 2= hel fac.båge

(Parod registreringar Se särskilt schema)

5 st Intraorala foton

6 st Extraorala foton

Röntgen panorama, datum………………………………………....

Röntgen profil, datum om den finns…………………………….

Röntgen apikalröntgen, datum om det finns…………………

CBCT, datum om det finns ………………………………………….

Remiss för friläggning (datum)……………………………………

::::::::::::::::::::::::::::::::::::::::::::::::::::::::::::::::::::::::::::::::::::::::::::::::::::::::::::::::::::::::::::::::::::::::::::

**# Senare registreringar**

Typ av friläggning………. 1 = med avl ben, 2 = endast friläggning

Friläggning, datum………………………………………………….

Tandens läge på Panorama:

Sektor ……………………………

Alpha vinkel …………………

Avstånd till ocklusionsplanet …………………….

:::::::::::::::::::::::::::::::::::::::::::::::::::::::::::::::::::::::::::::::::::::::::::::::::::::::::::::::::::::::::::::::::::::::::::::

**Behandlingsstart /ortodontiskt drag.**

Datum………………………….

**Behandlingsstart/ortodontiska drag ut till tandbågen**

Datum …………………….

**Hörntanden frambruten**

Datum. ………………………

Intraorala foton

Apikalröntgen eller Panorama, om det anses lämpligt

**Hörntanden på plats i tandbågen**

Datum…………………………...

Intraorala foton

Apikalröntgen eller Panorama, om lämplig

Parod registrering se särskilt schema

**Behandlingstid**, friläggning till behandlingen klar (månader)…………………………….

Under behandlingens gång tas erforderliga intraorala röntgen för att utvärdera dragriktning och eventuella låsningar. Även erforderliga panoramaröntgen tas under behandlingens gång.

I samband med oväntade tillstånd under behandlingen tas intraorala foton
