# Supplementary material for: Treatment of Palatally Displaced Canines in Children: A Randomized Controlled Pilot Trial on Exposure Time and Patient Perception of Two Closed Surgical Methods
Source: Clin Exp Dent Res. 2025 Oct 13;11(5):e70233. doi: 10.1002/cre2.70233 (PMC12516783; doi:10.1002/cre2.70233)
Supplement: Supplementary file 2 — supp. [file CRE2-11-e70233-s003.docx]

Enkät 3 Datum………………. . Patientnummer…………………….

Var snäll och gör ett litet streck på linjen efter frågan för att visa vad du tycker för tillfället. Om du instämmer helt med något av alternativen bör Du sätta krysset på det vågräta strecket.

1. Upplevde du smärta när gummitråden spändes för att flytta hörntanden?

| Ingen smärta |  |  |  |  | Outhärdlig smärta |
| --- | --- | --- | --- | --- | --- |
|  |  |  |  |  |  |
|  |  |  |  |  |  |

2. Upplevde Du smärta under tiden gummitråden drogs åt och knöts?

| Ingen smärta |  |  |  |  | Outhärdlig smärta |
| --- | --- | --- | --- | --- | --- |
|  |  |  |  |  |  |
|  |  |  |  |  |  |

3. Har Du tagit värktabletter/medicin mot smärtan idag?

Nej

Ja Vilken?

Hur många och vilken dos?

4. Upplevde Du obehag när gummitråden spändes för att flytta hörntanden?

| Ingen smärta |  |  |  |  | Outhärdlig smärta |
| --- | --- | --- | --- | --- | --- |
|  |  |  |  |  |  |
|  |  |  |  |  |  |

5. Upplevde Du obehag under tiden gummitråden drogs åt och knöts?

| Ingen smärta |  |  |  |  | Outhärdlig smärta |
| --- | --- | --- | --- | --- | --- |
|  |  |  |  |  |  |
|  |  |  |  |  |  |

6. Upplevde Du någon del av att gummitråden drogs åt och knöts obehaglig?

Ja i så fall vad……………………………………………………………………………

Nej

Bilaga 2(Enkät 3:1)

7. Har Du smärta efter att gummitråden spännts för att flytta hörntanden?

| Ingen smärta |  |  |  |  | Outhärdlig smärta |
| --- | --- | --- | --- | --- | --- |
|  |  |  |  |  |  |
|  |  |  |  |  |  |

8. Har Du haft obehag från området där gummitråden har spännts?

| Ingen smärta |  |  |  |  | Outhärdlig smärta |
| --- | --- | --- | --- | --- | --- |
|  |  |  |  |  |  |
|  |  |  |  |  |  |
