# Supplementary material for: Treatment of Palatally Displaced Canines in Children: A Randomized Controlled Pilot Trial on Exposure Time and Patient Perception of Two Closed Surgical Methods
Source: Clin Exp Dent Res. 2025 Oct 13;11(5):e70233. doi: 10.1002/cre2.70233 (PMC12516783; doi:10.1002/cre2.70233)
Supplement: Supplementary file 4 — supp. [file CRE2-11-e70233-s002.docx]

Enkät 1 Datum……………….. Patientnummer…………………….

Var snäll och gör ett litet streck på linjen efter frågan för att visa vad du tycker för tillfället. Om du instämmer helt med något av alternativen bör Du sätta krysset på det vågräta strecket.

1. Upplevde du smärta när Du fick injektion/bedövning?

| Ingen smärta |  |  |  |  | Outhärdlig smärta |
| --- | --- | --- | --- | --- | --- |
|  |  |  |  |  |  |
|  |  |  |  |  |  |

2. Upplevde Du smärta under operationen/friläggningen?

| Ingen smärta |  |  |  |  | Outhärdlig smärta |
| --- | --- | --- | --- | --- | --- |
|  |  |  |  |  |  |
|  |  |  |  |  |  |

3. Har Du tagit värktabletter/medicin mot smärtan idag?

Nej

Ja Vilken?

Hur många och vilken dos?

4. Upplevde Du obehag när du fick injektion/bedövning?

| Ingen smärta |  |  |  |  | Outhärdlig smärta |
| --- | --- | --- | --- | --- | --- |
|  |  |  |  |  |  |
|  |  |  |  |  |  |

5. Upplevde Du obehag under operationen/friläggningen?

| Ingen smärta |  |  |  |  | Outhärdlig smärta |
| --- | --- | --- | --- | --- | --- |
|  |  |  |  |  |  |
|  |  |  |  |  |  |

6. Upplevde Du någon del av operationen/friläggningen som obehaglig?

Ja i så fall vad……………………………………………………………………………

Nej

Bilaga 2(Enkät 1:1)

7. Har Du smärta efter operationen/friläggningen?

| Ingen smärta |  |  |  |  | Outhärdlig smärta |
| --- | --- | --- | --- | --- | --- |
|  |  |  |  |  |  |
|  |  |  |  |  |  |

8. Har Du obehag från området där operationen/friläggningen gjordes?

| Ingen smärta |  |  |  |  | Outhärdlig smärta |
| --- | --- | --- | --- | --- | --- |
|  |  |  |  |  |  |
|  |  |  |  |  |  |

Om Du har besvär efter operationen/friläggningen, hur mycket påverkar det

|  | Inga svårigheter | Vissa svårigheter | Svårt | Mycket  svårt | Enormt svårt |
| --- | --- | --- | --- | --- | --- |
| 9.Dina fritidsaktiviteter |  |  |  |  |  |
| 10.Talet |  |  |  |  |  |
| 11. Att ta ett stort bett |  |  |  |  |  |
| 12. Att tugga hård föda |  |  |  |  |  |
| 13.Att tugga mjuk mat |  |  |  |  |  |
| 14.Skolarbete |  |  |  |  |  |
| 15.Att dricka |  |  |  |  |  |

16.Att skratta

| 17.Att tugga mot motstånd |  |  |  |  |  |
| --- | --- | --- | --- | --- | --- |
| 18.Att gäspa |  |  |  |  |  |

Bilaga 2(Enkät 1:1)

Att äta mat innebär att ta ett bett, tugga och svälja. Hur svårt är det för Dig att äta

|  | Inga svårigheter | Vissa svårigheter | Svårt | Mycket svårt | Enormt svårt |
| --- | --- | --- | --- | --- | --- |
| 19.En hård smörgås |  |  |  |  |  |
| 20.Kött |  |  |  |  |  |
| 21.En rå morot |  |  |  |  |  |
| 22.En fralla |  |  |  |  |  |
| 23.Jordnötter |  |  |  |  |  |
| 24.Ett äpple |  |  |  |  |  |
| 25.en mjuk Kaka |  |  |  |  |  |

26. Har Du stannat hemma från skolan idag på grund av smärta efter operation/ friläggning?

Ja

Nej

27. Har Du avstått från fritidsaktiviteter idag på grund av smärta efter operation/ friläggning?

Ja I så fall vad……………………………………….

Nej
